# Temporal left ventricular ejection fraction variations and outcomes in wide population of cardiovascular patients with and without heart failure

**DOI:** 10.3389/fcvm.2025.1559258

**Published:** 2025-03-06

**Authors:** Radosław Szczerba, Wiktoria Żelazna, Jakub Sokołowski, Natalia Wyroba, Zuzanna Wydrych, Michał Wita, Małgorzata Cichoń, Michał Orszulak, Katarzyna Mizia-Stec, Krystian Wita, Maciej T. Wybraniec

**Affiliations:** ^1^ First Department of Cardiology, School of Medicine in Katowice, Medical University of Silesia, Katowice, Poland; ^2^ Upper-Silesian Medical Center, Katowice, Poland; ^3^ Member of the European Reference Network on Heart Diseases, ERN GUARD-HEART, Amsterdam, Netherlands

**Keywords:** heart failure with improved ejection fraction, HFimpEF, left ventricular ejection fraction, heart failure, mortality, MACCE

## Abstract

**Introduction:**

Heart failure (HF) with improved ejection fraction (HFimpEF) was shown to be related with improved outcome but increase of left ventricular ejection fraction (LVEF) in patients without HF is of less known clinical significance. The aim of the study was to evaluate long-term prognosis in patients with different cardiovascular disorders, with and without HF, depending on temporal variations of LVEF.

**Methods:**

The study covered 31 920 patients (median age 71 years, 37.7% females) with different cardiovascular disorders and at least two measurements of LVEF separated by ≥1 month. Clinical parameters were acquired from database of Academic Repository of Clinical Cases of Medical University of Silesia. HFimpEF was defined by LVEF increase ≥10% in HF patients in relation to baseline value. The endpoints were all-cause mortality and Major Adverse Cardiac and Cerebrovascular Event (MACCE).

**Results:**

The median follow-up time was 51.5 months and LVEF was measured median 2 times. HF was diagnosed in 12 152 patients (38.1%), of which 2 843 (23.4%) experienced HFimpEF. MACCE occurrence was greater in HF than non-HF patients (12.78%/year vs. 6.07%/year, *p* < 0.001). In patients with HF, Kaplan–Meier survival curves showed significantly lower MACCE occurrence in HFimpEF and stable LVEF than in decreased LVEF (11.46%/year vs. 12.5%/year vs. 21.6%/year; log-rank *p* = 0.199 and *p* < 0.001) and HFimpEF constituted one of independent predictors of MACCE (HR = 0.84, 95% CI: 0.76–0.93). Conversely, in non-HF population patients with LVEF improvement had higher MACCE occurrence than patients with stable LVEF and lower than deteriorating LVEF (6.97%/year vs. 5.72%/year vs. 14.55%/year respectively; log-rank *p* = 0.001 and *p* < 0.001).

**Conclusions:**

Temporal increase of LVEF corresponds with improved survival in patients with HF but not among non-HF patients.

## Introduction

Heart failure (HF) is a syndrome of symptoms and/or signs resulting in elevated intracardiac pressures and/or inadequate cardiac output at rest or during exercise (1). The 5-year mortality rate ranges from over 50% to almost 70% (2, 3).

There is no statistically significant difference in all-cause mortality and hospitalization rate between HF with reduced ejection fraction (HFrEF) and HF with preserved ejection fraction (HFpEF) (2, 3).

The meta-analysis by He et al. proved a significantly lower risk of hospitalization for HF and death among patients with improved LVEF in comparison to HFrEF and HFpEF patients (4). Due to that, a new group of HF patients has been established: heart failure with improved (HFimpEF) or recovered (HFrecEF) ejection fraction (5). Different studies used variable definitions over time (6). Valsartan Heart Failure Trial defined an improvement of LVEF as the increase of LVEF from baseline <35% to >40% in follow-up measurement after 12 months (7). Meta-analysis of Jorgensen et al. described the improvement of LVEF as the recovery of LVEF by >5% in the follow-up measurement after median time of 19 months (8). Another definition was brought in the study which used the Swedish Heart Failure Registry and where improvement of LVEF was defined as an upgrade in HF subtype (from HFrEF to HFmrEF, or HFrEF/HFmrEF to HFpEF) (9). In the cluster analysis presented by A. Perry et al. LVEF improvement was defined as the increase of LVEF from <35% to >50% (10). According to the latest consensus the improvement of LVEF was established as a baseline LVEF of ≤40% and an increase of ≥10% and a follow-up measurement of >40% (11). Although HFimpEF has been linked to favorable survival, no study has so far studied LVEF temporal variations in a large cohort of HF and non-HF patients with different cardiac conditions. The aim of the present study was to evaluate long-term prognosis in a wide spectrum of patients with cardiovascular disorders with and without HF depending on temporal variations of LVEF.

## Materials and methods

### Study population

The study covered 31,920 adult patients with different cardiovascular disorders hospitalized in Upper-Silesian Medical Center or University Clinical Center between February 2018 and October 2023. Inclusion criteria were at least 2 LVEF measurements separated by at least 1 month.

Data was gathered using the Academic Repository of Clinical Cases of the Medical University of Silesia in Katowice, consisting of all patients' data collected across three medical centers (Upper-Silesian Medical Center, University Clinical Center, Upper-Silesian Child Health Center) located in Katowice, Poland.

### Data collection and study endpoints

**Figure 1:**
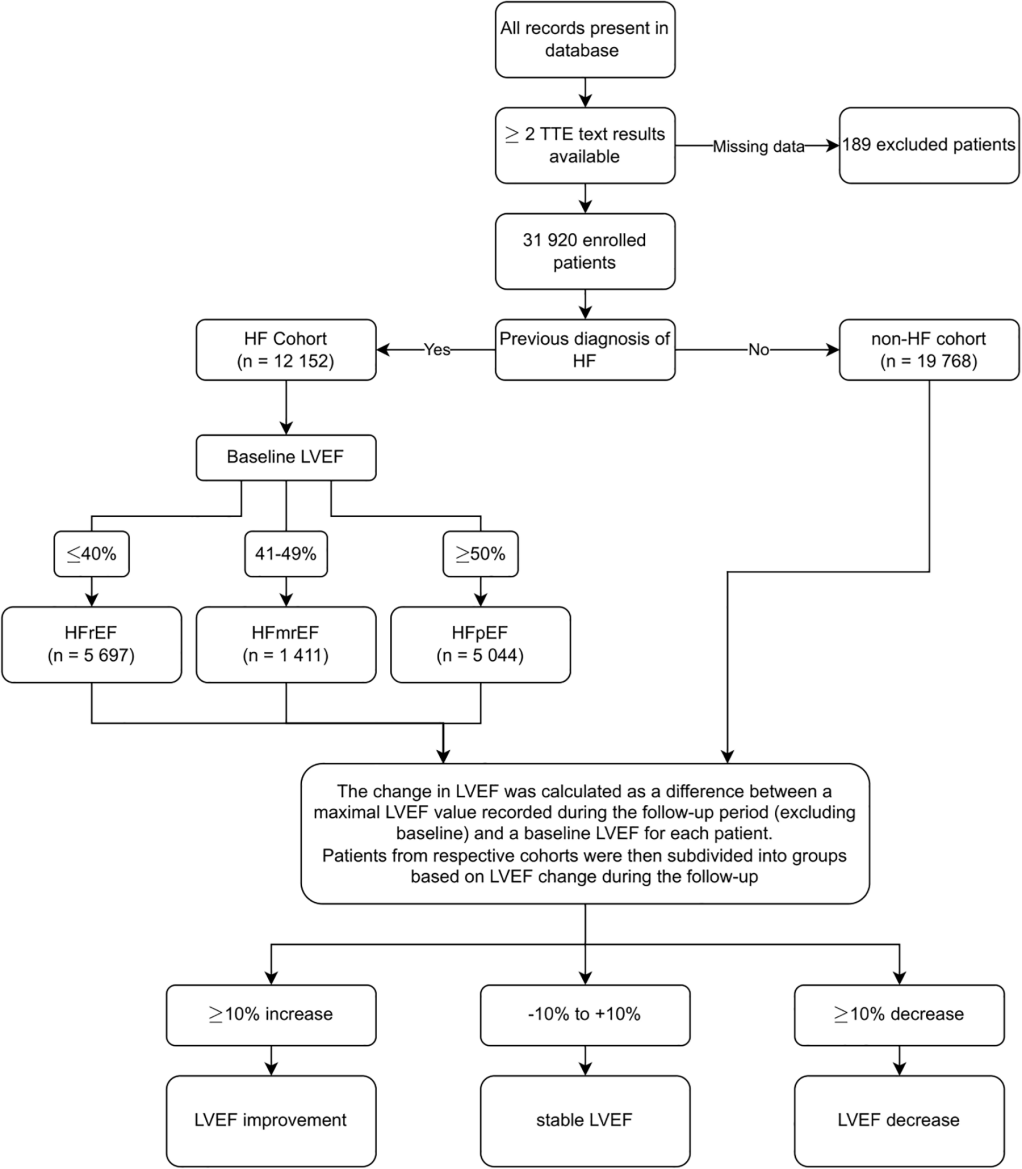
Methodology flowchart. TTE, transthoracic echocardiography; HF, heart failure; LVEF, left ventricular ejection fraction; HFrEF, heart failure with reduced ejection fraction; HFmrEF, heart failure with mildly reduced ejection fraction; HFpEF, heart failure with preserved ejection fraction.

The analysis included data on demographic parameters, comorbidities, echocardiography text results, laboratory test results, all-cause mortality, non-fatal myocardial infarction, stroke. Data on comorbidities were drafted from patients' electronic health records, discharge summaries and hospitals' system-coded diagnoses combined. LVEF measurements were extracted from digitally stored echocardiography text results using text mining methods and were checked manually for correctness of this extraction. Exclusion criteria comprised missing data on baseline and/or follow-up LVEF which led to exclusion of 189 patients. Detailed data on methodology is presented on a flow-chart diagram (Figure 1).

For all the LVEF measurements available both in the 2-chamber and 4-chamber plane, mean value was calculated instead. The CHA_2_DS_2_-VASc score was calculated based on acquired information for all the patients. Glomerular filtration rate (eGFR) was estimated using the MDRD simplified formula for all patients with measured serum creatinine levels (*n* = 29 390, 92.1%). The primary endpoint was all-cause mortality confirmed by the national healthcare provider, expressed as % per year. The secondary endpoint was Major Adverse Cardiac and Cerebrovascular Event (MACCE) occurring at least 7 days subsequent to the first LVEF measurement.

### Definitions

HF was diagnosed in patients who presented symptoms and/or signs of HF (e.g., breathlessness, fatigue, ankle swelling) coexisting with confirmed LVEF <50%. In patients with LVEF ≥50%, symptoms and/or signs of HF had to be accompanied by objective evidence of cardiac dysfunction, including N-terminal pro-B type natriuretic peptide ≥125 pg/ml, B type natriuretic peptide ≥35 pg/ml or structural abnormalities, such as LA volume index >32 ml/m^2^, mitral E velocity >90 cm/s, septal e' velocity <9 cm/s, E/e’ ratio >9, LV mass index ≥95 g/m^2^ among females, ≥115 g/m^2^ among males, relative wall thickness >0.42.

HF cohort consists of patients with previously recorded diagnosis of I50. Patients without this diagnosis constitute the non-HF cohort. Heart failure with reduced ejection fraction (HFrEF) was defined as LVEF ≤40%. Heart failure with mildly reduced ejection fraction (HFmrEF) was defined as LVEF between 41% and 49%. Heart failure with preserved ejection fraction (HFpEF) was defined as LVEF ≥50% with evidence of structural and/or functional cardiac abnormalities and/or raised natriuretic peptides. In patients with AF, if the diagnosis of HF was based only on natriuretic peptides, higher threshold of NT-proBNP level >365 pg/ml was applied (1).

LVEF variations were calculated as a difference between maximal LVEF measured during follow-up, at least one month after initial examination, and the baseline value. In case of multiple follow-up measurements available, the highest value of follow-up LVEF was chosen for calculations. LVEF fluctuations were categorized into (a) LVEF improvement in case of LVEF increase by ≥10%, (b) stable LVEF in case of ΔLVEF from −10% to +10% and (c) LVEF decrease in case of LVEF decrease by ≥10% in comparison to initial value. HFimpEF was defined as a ≥10% increase of LVEF related to baseline measurement in patients with the diagnosis of HF, while it was particularly investigated in subpopulation of patients with initial HFrEF given formerly published documents (11).

MACCE was defined as the first occurrence of one of the following events: (1) all-cause death, (2) non-fatal myocardial infarction, (3) ischemic stroke.

The study was approved by the Ethics Committee of Medical University of Silesia and complied with the Declaration of Helsinki and was exempt from informed consent given retrospective, anonymous and registry-based design (BNW/NWN/0052/KB/127/24).

### Statistical analysis

Continuous data were reported as medians with 25th–75th percentiles or means with standard deviations while categorical data were presented as absolute numbers (*n*) with percentages (%). Differences between study groups were assessed using the Mann–Whitney U test for continuous variables and *χ*^2^ test for categorical variables. The multivariate Cox proportional hazards model was used to assess predictors of all-cause mortality and MACCE. The multivariate logistic regression model was used to assess predictors of LVEF improvement. Both models were carried out using a stepwise approach. All the variables with *p* < 0.1 were included in the regression analysis. Kaplan–Meier curves and log-rank test were used to compare survival between HF and non-HF as well as HFimpEF and non-HFimpEF groups with endpoints being respectively all-cause death and MACCE for both groups. All the results were considered statistically significant at two-sided *p*-value < 0.05.

## Results

### General description of the study group

General characteristics of the study population were presented in Table 1. A total of 31,920 patients with a mean age of 69.3 ± 12.9 years met the inclusion criteria. The median time of observation was 51.5 months (28.8; 65.4). Females accounted for 37.7% of the study population. HF was diagnosed in 12,152 (38.1%) patients. Among them, 46.9% (*n* = 5,697) fell into the category of HFrEF, 11.6% (*n* = 1,211) had HFmrEF and 41.5% (*n* = 5,044) had HFpEF.

**Table 1:** Comparison between non-HF patients (*n* = 19,768) and HF patients (*n* = 12,152) - qualitative and quantitative variables.

Variable		Study population	Non-HF patients	HF patients	*P*-value
		*n* (%) or mean ± SD	*n* (%) or mean ± SD	*n* (%) or mean ± SD	
Sex (female)		12,046 (37.7)	8,211 (41.5)	3,835 (31.6)	<0.001
Age [years]		69.3 ± 12.9	67.9 ± 13.5	71.5 ± 11.6	<0.001
Number of LVEF measurements [*n*]		3.2 ± 1.9	2.8 ± 1.4	3.7 ± 2.5	<0.001
Baseline LVEF [%]		49.2 ± 12.2	53.6 ± 8.2	42 ± 14.1	<0.001
Mean LVEF [%]		49.0 ± 11.2	53.5 ± 7.1	41.7 ± 12.7	<0.001
Mean LVEF in follow-up [%]		49.0 ± 11.5	53.5 ± 7.6	41.8 ± 13.1	<0.001
10% LVEF increase [*n*]		5,232 (16.4)	2,389 (12.1)	2,843 (23.4)	<0.001
ΔLVEF vs. baseline [%]		2.0 ± 8.2	1.3 ± 6.9	3.2 ± 9.8	<0.001
AF		9,109 (28.5)	4,706 (23.8)	4,403 (36.2)	<0.001
HA		21,509 (67.4)	12,996 (65.8)	8,513 (70.1)	<0.001
DM		9,688 (30.4)	5,083 (25.7)	4,605 (37.9)	<0.001
Dyslipidemia		3,089 (9.7)	2,047 (10.4)	1,042 (8.6)	<0.001
IHD		11,298 (35.4)	7,054 (35.7)	4,244 (34.9)	0.168
CKD		1,390 (4.4)	555 (2.8)	835 (6.9)	<0.001
MI		8,822 (27.6)	5,020 (25.4)	3,802 (31.3)	<0.001
Hb [g/dl]		12.4 ± 2.4	12.1 ± 2.2	12.7 ± 2.5	0.138
SCr [mg/dl]		1.1 ± 0.7	1.0 ± 0.6	1.2 ± 0.9	<0.001
eGFR [ml/min/1.73 m^2^]		72.1 ± 29.3	75.4 ± 28.8	67.1 ± 29.3	<0.001
CHA_2_DS_2_-VASc score		3.2 ± 1.6	2.8 ± 1.5	4 ± 1.5	<0.001
MACCE	Total	3,969 (12.4)	1,731 (8.8)	2,238 (18.4)	<0.001
	HFrEF	1,168 (3.7)		1,168 (9.6)	<0.001
	HFmrEF	233 (0.7)		233 (1.9)	
	HFpEF	837 (2.6)		837 (6.9)	
Death	Total	2,091 (6.6)	710 (3.6)	1,381 (11.4)	<0.001
	HFrEF	782 (2.4)		782 (6.4)	<0.001
	HFmrEF	133 (0.4)		133 (1.1)	
	HFpEF	466 (1.4)		466 (3.8)	
First LVEF measurement to MACCE [days]		528.4 ± 540.8	529.9 ± 556.5	527.3 ± 528.4	<0.109
First LVEF measurement to death [days]		587.3 ± 557.7	535.3 ± 571	612.3 ± 549.7	<0.001

LVEF, left ventricular ejection fraction; Baseline LVEF, first measurement of LVEF; Mean LVEF, mean value of LVEF across all measurements; Mean LVEF in follow-up, mean value of LVEF across all measurements excluding baseline; AF, atrial fibrillation; HA, arterial hypertension; DM, diabetes mellitus; IHD, ischemic heart disease; CKD, chronic kidney disease; MI, myocardial infarction; Hb, hemoglobin; SCr, serum creatinine; eGFR, estimated glomerular filtration rate; MACCE, major adverse cardiac and cerebrovascular event.

The mean number of LVEF measurements was 3.2 ± 1.9. During the follow-up period, 16.4% (*n* = 5,232) of patients experienced maximal LVEF improvement of at least 10%, which was classified as HFimpEF in 2,843 HF patients (8.9% of the entire study population; 23.4% of HF patients). Baseline LVEF of the population was 49.2% ± 12.2, with the mean LVEF of 49% ± 11.2 across all measurements (including baseline) and mean LVEF in follow-up (excluding baseline) of 49% ± 11.5%.

Arterial hypertension (HA) was the most common comorbidity, affecting 67.4% (*n* = 21,509) of the patients, followed by diabetes mellitus (DM) at 30.4% (*n* = 9,688) and myocardial infarction (MI) at 27.6% (*n* = 8,822).

MACCE occurred in 12.4% (8.55%/year; *n* = 3,969) of the patients, and the mortality rate was 6.6% (4.1%/year; *n* = 2 091). The median time from the first LVEF measurement to MACCE was 331 (40; 925) days.

#### Comparison between HF and non-HF patients

A complete comparison between non-HF and HF patients is shown in Table 1. HF was diagnosed in 12,152 (38.1%) patients of the whole study cohort. Among them, 68.4% (*n* = 8,317) were males and 31.6% (*n* = 3,835) were females. In 23.4% (*n* = 2,843) of the HF group, a 10% increase in LVEF has been noted. The mean age was higher in the group of patients with HF (71.5 ± 11.6) than in non-HF patients (67.9 ± 13.5) with a *p* < 0.001.

Baseline LVEF was notably lower in HF patients, averaging 42% ± 14.1 compared to 53.6% ± 8.2 in non-HF patients, with *p* < 0.001. Similarly, the mean LVEF across all measurements was lower in HF patients, recorded at 41.7% ± 12.7 compared to 53.5% ± 7.1 in non-HF patients, with a *p* < 0.001. During follow-up, HF patients continued to exhibit lower LVEF values, with a mean of 41.8% ± 13.1 compared to 53.5% ± 7.6 in non-HF patients (*p* < 0.001). The number of LVEF measurements was higher among HF patients, averaging 3.7 ± 2.5 compared to 2.8 ± 1.4 in non-HF patients.

Mortality in HF patients (11.4%; 6.82%/year; *n* = 1,381) was significantly higher than in non-HF patients (3.6%; 2.45%/year%; *n* = 710). A complete comparison between survivors and non-survivors within HF cohort is shown in Table 2. The incidence of MACCE was also higher in the HF group at 18.4% (12.78%/year, *n* = 2,238) compared to 8.8% of patients without HF (6.07%/year, *n* = 1,731).

**Table 2:** Comparison between survivors (*n* = 10,771) and patients who died on follow-up (*n* = 1,381) in HF group (*n* = 12,152) - qualitative and quantitative variables.

Variable		HF-survivors	HF- non-survivors	*P*-value
		*n* (%) or mean ± SD	*n* (%) or mean ± SD	
Sex (female)		3,350 (31.1)	485 (35.1)	0.002
Age [years]		71.3 ± 11.7	73.6 ± 10.9	<0.001
HF class	HFrEF	4,915 (45.6)	782 (56.6)	<0.001
	HFmrEF	1,278 (11.9)	133 (9.6)	
	HFpEF	4,578 (42.5)	466 (33.7)	
Number of LVEF measurements [*n*]		3.7 ± 2.3	4.2 ± 3.2	<0.001
Baseline LVEF [%]		42.3 ± 14	39 ± 14.6	<0.001
Mean LVEF [%]		42.4 ± 12.5	36.6 ± 13.5	<0.001
Mean LVEF in follow-up [%]		42.6 ± 12.7	35.7 ± 14.4	<0.001
10% LVEF increase [*n*]		2,578 (23.9)	265 (19.2)	<0.001
ΔLVEF vs. baseline [%]		3.5 ± 9.6	0.8 ± 10.9	<0.001
AF		3,865 (35.9)	538 (39)	0.025
HA		7,539 (70)	974 (70.5)	0.69
DM		3,967 (36.8)	638 (46.2)	<0.001
Dyslipidemia		963 (8.9)	79 (5.7)	<0.001
IHD		3,850 (35.7)	394 (28.5)	<0.001
CKD		670 (6.2)	165 (11.9)	<0.001
Hb [g/dl]		12.5 ± 2.4	13.5 ± 2.9	0.283
SCr [mg/dl]		1.2 ± 0.8	1.4 ± 1	<0.001
eGFR [ml/min/1.73 m^2^]		68.2 ± 28.8	58.2 ± 31.9	<0.001
CHA_2_DS_2_-VASc score		4 ± 1.5	4.3 ± 1.5	<0.001

HF, heart failure; HFrEF, heart failure with reduced ejection fraction; HFmrEF, heart failure with mildly reduced ejection fraction; HFpEF, heart failure with preserved ejection fraction; LVEF, left ventricular ejection fraction; Baseline LVEF, first measurement of LVEF; Mean LVEF, mean value of LVEF across all measurements; Mean LVEF in follow-up, mean value of LVEF across all measurements excluding baseline; AF, atrial fibrillation; HA, arterial hypertension; DM, diabetes mellitus; IHD, ischemic heart disease; CKD, chronic kidney disease; Hb, hemoglobin; SCr, serum creatinine; eGFR, estimated glomerular filtration rate.

### Kaplan–Meier survival curves – mortality and MACCE in HF and non-HF cohort

**Figure 2:**
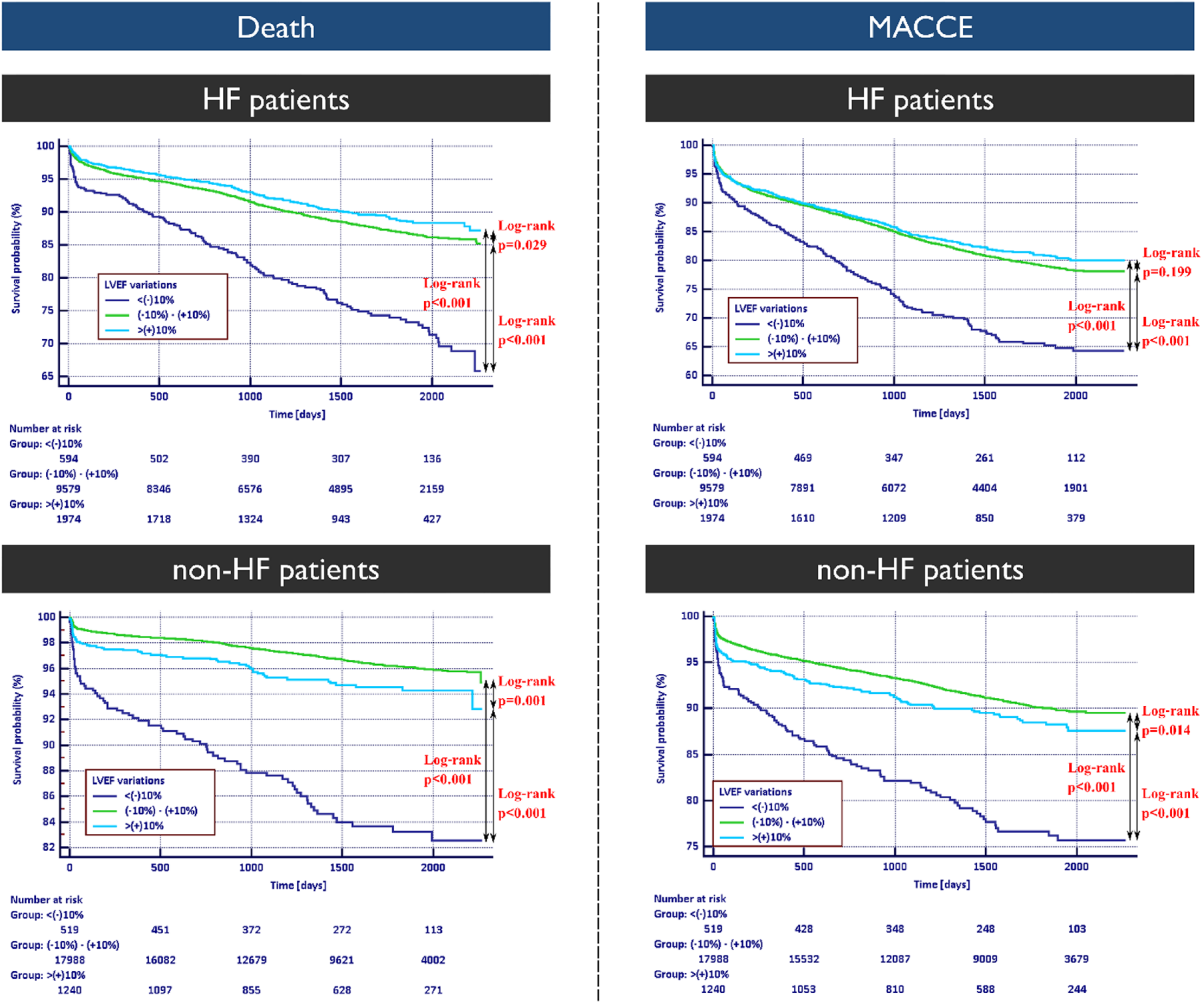
Kaplan–Meier survival curves for death and major adverse cardiac and cerebrovascular events occurrence in patients with and without heart failure diagnosis with respective log-rank test results. HF, heart failure; MACCE, major adverse cardiac and cerebrovascular event.

In the HF cohort, the study showed a lower rate of death in patients with improved LVEF in comparison to patients with stable and decreased LVEF (9.1% vs. 11.0% vs. 24.5% respectively; 5.45%/year vs. 6.59%/year vs. 14.67%/year; log-rank *p* = 0.029 and *p* < 0.001). The analysis revealed that patients with improved LVEF had a comparable rate of MACCE to patients with stable LVEF and lower than patients with decreasing LVEF (16.5% vs. 18.0% vs. 31.1% respectively; 11.46%/year vs. 12.5%/year vs. 21.6%/year; log-rank *p* = 0.199 and *p* < 0.001) (Figure 2).

Non-HF cohort with LVEF improvement had higher risk of mortality than patients with stable LVEF and lower than patients with decreasing LVEF (4.8% vs. 3.2% vs. 15.0% respectively;, 3.27%/year vs. 2.18%/year vs. 10.2%/year respectively; log-rank *p* = 0.001 and *p* < 0.001). The rate of MACCE onset in non-HF patients with improved LVEF was higher than in patients with stable LVEF and lower than in patients with decreasing LVEF (10.1% vs. 8.3% vs. 21.1% respectively; 6.97%/year vs. 5.72%/year vs. 14.55%/year respectively; log-rank *p* = 0.001 and *p* < 0.001) (Figure 2).

**Figure 3:**
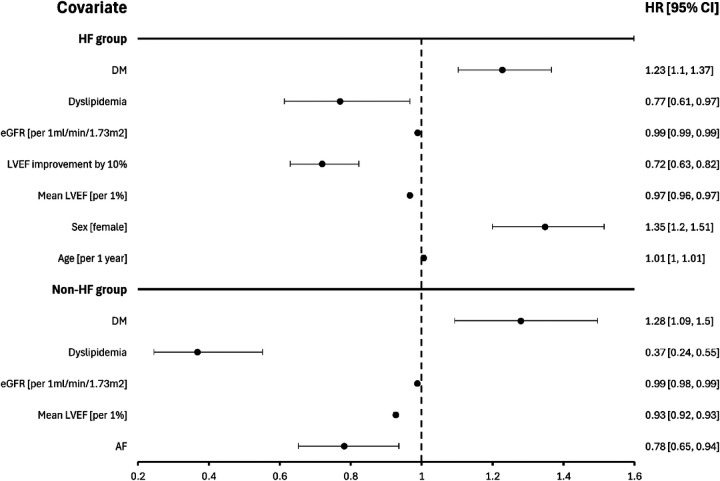
Predictors of mortality in patients with and without heart failure. DM, diabetes mellitus; eGFR, estimated glomerular filtration rate; LVEF, left ventricular ejection fraction.

In the population of patients with HF (*n* = 12,152) Cox proportional hazard model (Figure 3) shows that survival was independently associated with LVEF increase by 10%, DM, female sex, and age.

In the population of patients without HF (*n* = 19,768) Cox proportional hazard model (Figure 3) shows that survival was independently associated with DM, dyslipidemia, eGFR, mean LVEF and AF.

Predictors for MACCE occurrence in those cohorts are presented in Supplementary Figure 1.

### Kaplan–Meier survival curves for death and MACCE endpoints in HF subpopulations

**Figure 4:**
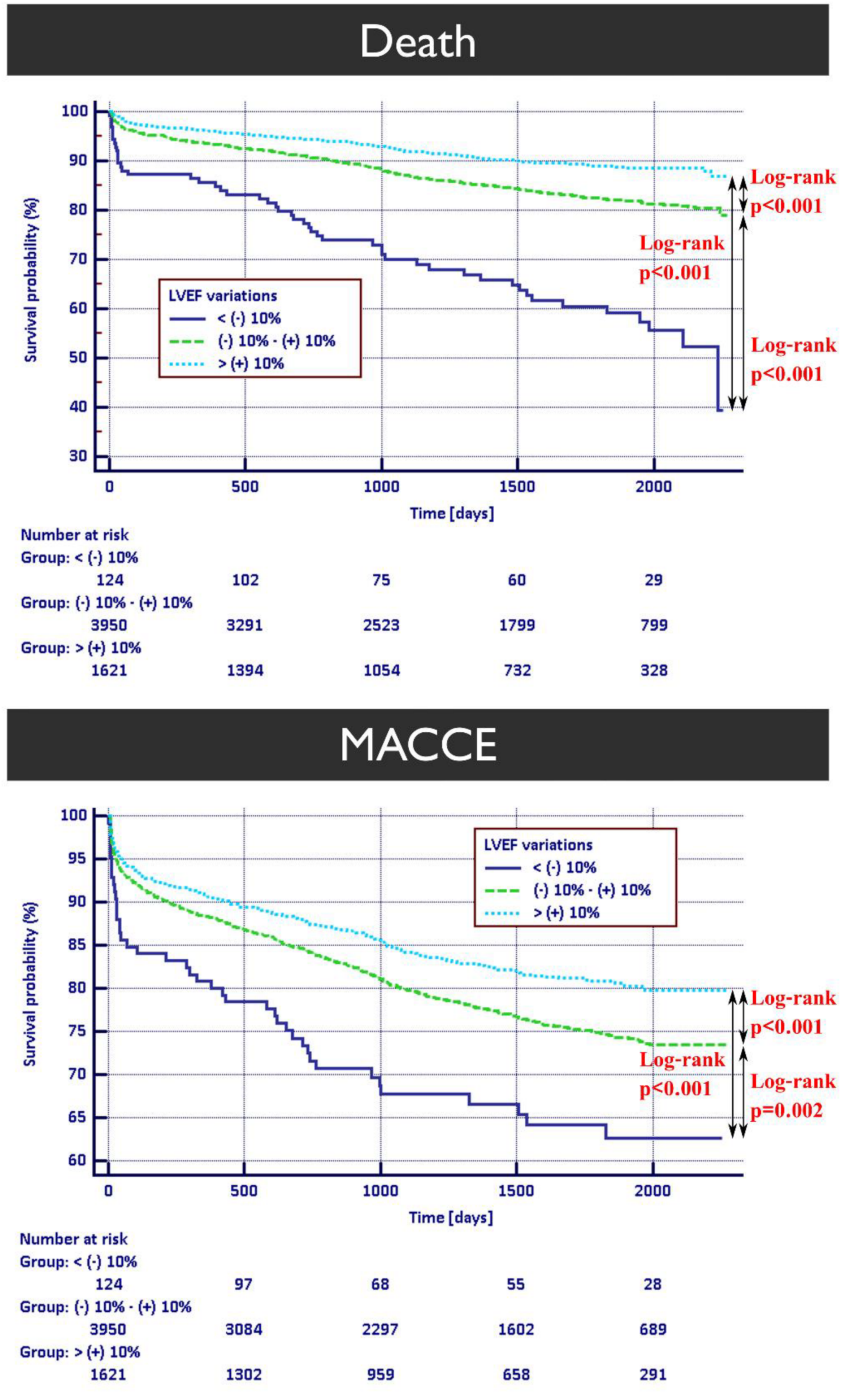
Kaplan–Meier survival curves for death and MACCE occurrence in patients with heart failure with reduced ejection fraction. HFrEF, heart failure with reduced ejection fraction; MACCE, major adverse cardiac and cerebrovascular event.

In patients with HFrEF (Figure 4) survival probability with death as an endpoint was the highest in the group with LVEF improvement. HFmrEF patients (Supplementary Figure 2) did not exhibit significant changes in survival between stable and improvement subgroups. Finally, in HFpEF patients (Supplementary Figure 3), stable LVEF was associated with better prognosis in relation to other subgroups in terms of all-cause mortality.

In both HFrEF and HFmrEF patients Kaplan–Meier survival curves for MACCE endpoint show similar trend favoring individuals with improved LVEF. Contrary to those findings, HFpEF cohort exhibits better prognosis in patients with stable LVEF.

### Predictors of mortality in HF subpopulations

**Figure 5:**
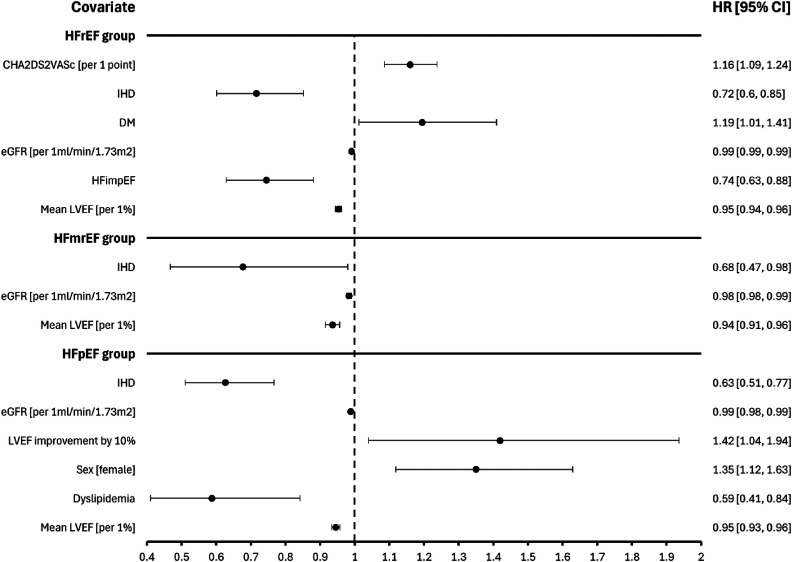
Predictors of mortality depending on the initial diagnosis of heart failure with reduced or mildly reduced or preserved ejection fraction. CI, confidence interval; IHD, ischemic heart disease, DM, diabetes mellitus; eGFR, estimated glomerular filtration rate; HFrEF, heart failure with reduced ejection fraction; HFmrEF, heart failure with mildly reduced ejection fraction; HFpEF, heart failure with preserved ejection fraction; HFimpEF, heart failure with improved ejection fraction; LVEF, left ventricular ejection fraction.

In the population of patients with HFrEF (*n* = 5,593) Cox proportional hazard model (Figure 5) shows that survival was independently associated with HFimpEF, CHA_2_DS_2_-VASc and DM. In the population with HFmrEF (*n* = 1,358) survival was independently associated with IHD, eGFR and mean LVEF. Finally, in the HFpEF cohort (*n* = 4,844) survival was independently associated with LVEF increase by 10% and female sex.

### Predictors of LVEF improvement within the HFrEF subclass – logistic regression analysis

In the population of patients with HFrEF (*n* = 5,593) logistic regression analysis (Supplementary Figure 4) shows that LVEF improvement was independently associated with female sex, AF, HA, CKD and age.

### HFimpEF vs. non-HFimpEF patients in a HFrEF cohort

The comparison between HFimpEF and non-HFimpEF patients in HFrEF cohort was presented in Table 3. LVEF improved in 37% (*n* = 2,110) out of all people with HFrEF (*n* = 5,697). Among them 27.4% (*n* = 579) were females and 72.6% (*n* = 1,531) were males. The mean age was higher in the group of non-HFimpEF (71.3 ± 10.9) than in HFimpEF patients (69.4 ± 12.3).

**Table 3:** Comparison of different clinical variables and prognosis in subgroup of patients with baseline heart failure with reduced ejection fraction depending on the onset of heart failure with improved ejection fraction.

Variable	Non-HFimpEF patients	HFimpEF patients	*P*-value
	*n* = 3,587	*n* = 2,110	
	*n* (%) or mean ± SD	*n* (%) or mean ± SD	
Sex (female)	729 (20.3)	579 (27.4)	<0.001
Age [years]	71.3 ± 10.9	69.4 ± 12.3	<0.001
Number of LVEF measurements [*n*]	3.6 ± 2.4	4.5 ± 2.9	<0.001
Baseline LVEF [%]	30.1 ± 7.7	27.3 ± 8.8	0.881
Mean LVEF [%]	28.9 ± 7.8	34.8 ± 8.8	<0.001
Mean LVEF in follow-up [%]	28.6 ± 8.6	38.5 ± 10	<0.001
ΔLVEF vs. baseline [%]	0.8 ± 5.5	17.3 ± 7.6	<0.001
AF	1,182 (33)	850 (40.3)	<0.001
HA	2,252 (62.8)	1,380 (65.4)	0.048
DM	1,420 (39.6)	787 (37.3)	0.087
Dyslipidemia	192 (5.4)	134 (6.4)	0.117
IHD	1,086 (30.3)	669 (31.7)	0.259
CKD	195 (5.4)	158 (7.5)	0.002
MI	1,343 (37.4)	819 (38.8)	0.302
Ischemic stroke	115 (3.2)	82 (3.9)	0.175
Hb [g/dl]	13.2 ± 3	13 ± 2.3	0.043
SCr [mg/dl]	1.3 ± 0.8	1.2 ± 1	0.019
eGFR [ml/min/1.73 m^2^]	65.5 ± 33.6	66.9 ± 33	0.329
CHA_2_DS_2_-VASc score	3.8 ± 1.4	3.7 ± 1.5	<0.001
MACCE	815 (22.7)	353 (16.7)	<0.001
Death	584 (16.3)	198 (9.4)	<0.001

LVEF, left ventricular ejection fraction; Baseline LVEF, first measurement of LVEF; Mean LVEF, mean value of LVEF across all measurements; Mean LVEF in follow-up, mean value of LVEF across all measurements excluding baseline; AF, atrial fibrillation; HA, arterial hypertension; DM, diabetes mellitus; IHD, ischemic heart disease; CKD, chronic kidney disease; MI, myocardial infarction; Hb, hemoglobin; SCr, serum creatinine; eGFR, estimated glomerular filtration rate; MACCE, major adverse cardiac and cerebrovascular event.

The number of LVEF measurements was lower among non-HFimpEF patients averaging 3.6 ± 2.4 compared to 4.5 ± 2.9 among HFimpEF patients. The cohort diagnosed with HFimpEF had a higher mean value of LVEF across all measurements (34.8% ± 8.8) and across all measurements excluding baseline LVEF (38.5% ± 10) than the non-HFimpEF cohort (respectively 28.9% ± 7.8 and 28.6% ± 8.6).

The incidence of AF was greater in HFimpEF patients (40.3%, *n* = 850) than in non-HFimpEF patients (33%, *n* = 1,182). HA was diagnosed in 65.4% (*n* = 1,380) of the HFimpEF cohort and 62.8% (*n* = 2,252) of the non-HFimpEF cohort. CKD occurred in 5.4% (*n* = 195) of the non-HFimpEF group and 7.5% (*n* = 158) of the HFimpEF group.

Mortality in non-HFimpEF patients (16.3%; 9.76%/year; *n* = 584) was significantly higher than in HFimpEF patients (9.4%; 5.63%/year *n* = 198). Additionally, the non-HFimpEF cohort had a higher incidence of MACCE (22.7%; 15.76%/year *n* = 815) than HFimpEF (16.7%; 11.6%/year; *n* = 353).

## Discussion

To the best of our knowledge, this study is so far the first analysis to investigate both MACCE occurrence and all-cause mortality, based on temporal variations in LVEF among a substantial cohort of patients with different cardiac conditions, including both HF and non-HF cases. Patients with HF who showed improvement in LVEF experienced significantly lower mortality rates compared to those with stable or decreased LVEF. However, among non-HF individuals, those with improved LVEF had a higher mortality rate compared to patients with stable LVEF but still lower than those with decreasing LVEF. This comparison highlights that the clinical significance of LVEF improvement differs between HF and non-HF patients, with HF patients deriving more pronounced benefits from improved LVEF.

Our findings are in accordance with previous studies that have established LVEF improvement as a key prognostic marker in HF-patients (4, 7, 12–14). In the subanalysis of Val-HeFT trial, improvement of LVEF >40% within 12 months identified patients with improved 12-month survival in comparison to patients who had stable LVEF within the class of HFrEF (7). In the meta-analysis by Jorgensen et al, even a small increase of LVEF by >5% heralded reduced risk of death (5.8% vs. 17.5%, *p* < 0.001) (8). In the data based on Swedish Heart Failure Registry, about one in four patients with HFrEF experienced improvement of LVEF to HFmrEF and HFpEF category, which was linked to improved survival in comparison to stable LVEF (HR 0.62, 95%CI: 0.55–0.69) (9). Similar results were provided by Strange et al. who described fluctuations in LVEF in a cohort of 117,275 adults who have had at least 2 echocardiograms within 6 months. This study found that individuals being investigated for HF with decreased LVEF levels were linked to greatly increased cardiovascular-related and all-cause mortality with improvement of LVEF heralding better prognosis (15). Similarly, in our study, 37% of HFrEF patients improved LVEF by at least 10% and this was linked to decreased risk of death (9.4 vs. 16.3%, *p* < 0.001). Interestingly, the risk related with mild LVEF impairment in non-HF patients with LVEF >50% was greater in women than men, suggesting sex-based differences in the impact of LVEF on mortality (16). The present analysis further expands these observations to include the effect of LVEF variations on MACCE occurrence among non-HF cohort.

This study indicates that LVEF improvement in HFrEF cohort was predicted by female sex, presence of AF, chronic kidney disease, arterial hypertension and younger age (Supplementary Figure 4). These results are consistent with the former studies in the field (7–9), nevertheless, our study did not indicate non-ischemic etiology of HF as a predictor of survival.

## Study limitations

The study's findings are limited mainly by the retrospective nature of the gathered data and the large sample size. It should be noted that all the diagnoses were established in two medical centers being part of the Academic Repository. Any diagnoses and/or laboratory tests performed in a different medical center could be missed by a patient or the attending physician and therefore were not listed in patients' history or discharge summary resulting in missing data in the repository.

As there is no way to distinguish between missing information and a patient without a previous diagnosis of the disease, data on certain conditions could be underestimated which impacts the study's results. One of the conditions possibly affected by this fact was the diagnosis of dyslipidemia which could be overlooked especially in patients with multimorbidity leading to presumably lowered health risk associated with this diagnosis. For the same reason, the CHA_2_DS_2_-VASc score could be miscalculated for patients with missing data.

Records of NT-proBNP levels, although available for initial examination of HF patients by respective physicians, were not accessible in repository database. Furthermore, due to the Academic Repository limitations related to the method of collecting and organizing information in medical centers, the data on therapy could not be gathered with sufficient care for its quality.

Considering LVEF changes in time as the main focus area of this study it is important to note that the measurement of LVEF is a subjective procedure performed by a number of different physicians therefore being prone to error. Furthermore, not all of the patients had the same follow-up time resulting in a need for data censoring during survival analysis.

## Conclusions

LVEF variability is prevalent in patients with various cardiovascular disorders and in patients with a diagnosis of HF. In the non-HF group, patients with stable LVEF share a better prognosis than patients with LVEF variability (increase or decrease). In HF patients, onset of HFimpEF heralds improved prognosis with differences between subclasses. Improvements of LVEF in patients with HFrEF and HFmrEF determine better survival and decreased MACCE occurrence.

## Data Availability

The raw data supporting the conclusions of this article will be made available by the authors, without undue reservation.

## Supplementary material

Supplementary Figure 1 Predictors of MACCE in HF and non-HF patients. CI, confidence interval; DM, diabetes mellitus; eGFR, estimated glomerular filtration rate; LVEF, left ventricular ejection fraction; IHD, ischemic heart disease; AF, atrial fibrillation.

Supplementary Figure 2 Kaplan–Meier survival curves for death and MACCE occurrence in patients with HFmrEF with log-rank test result. HFmrEF, heart failure with mildly reduced ejection fraction; MACCE, major adverse cardiac and cerebrovascular event.

Supplementary Figure 3 Kaplan–Meier survival curves for death and MACCE occurrence in patients with HFpEF with log-rank test result. HFpEF, heart failure with preserved ejection fraction; MACCE, major adverse cardiac and cerebrovascular event.

Supplementary Figure 4 Predictors of heart failure with improved ejection fraction in patients with heart failure with reduced ejection fraction at baseline. CI, confidence interval; AF, atrial fibrillation; HA, arterial hypertension; CKD, chronic kidney disease.
